# Self-powered temperature-changing system driven by wind energy

**DOI:** 10.1038/s41378-024-00741-1

**Published:** 2024-09-26

**Authors:** Jiayu Li, Boxun Liu, Mingyang Li, Yahui Li, Wangyang Ding, Guanlin Liu, Jun Luo, Nan Chen, Lingyu Wan, Wenjuan Wei

**Affiliations:** 1 Center on Nanoenergy Research, Institute of Science and Technology for Carbon Peak & Neutrality, Guangxi University, Nanning, 530004 China; 2 State Key Laboratory of Featured Metal Materials and Life-cycle Safety for Composite Structures, School of Physical Science & Technology, Guangxi University, Nanning, 530004 China; 3 Department of Chemistry and the Tsinghua Center for Frontier Polymer Research, Tsinghua University, Beijing, 100084 PR China; 4 State Key Laboratory of Pollution Control and Resource Reuse, School of the Environment, Nanjing University, Nanjing, 210023 China; 5 Department of Micro/Nano Electronics, School of Electronic Information and Electrical Engineering, Shanghai Jiao Tong University, Shanghai, 200240 PR China; 6 State Key Laboratory of Featured Metal Materials and Life-cycle Safety for Composite Structures, MOE Key Laboratory of New Processing Technology for Nonferrous Metals and Materials, and School of Resources, Environment and Materials, Guangxi University, Nanning, 530004 China

**Keywords:** Electronic properties and materials, Electronic devices

## Abstract

Research on outdoor, mobile, and self-powered temperature-control devices has always been highly regarded. These devices can reduce energy consumption for cooling and heating, and they have broad market prospects. On this basis, a rotary disc-shaped triboelectric nanogenerator (TENG) with a maximum open-circuit voltage of 6913 V, a maximum short-circuit current of 85 μA, and a maximum transferred charge of 1.3 μC was prepared. We synthesized a ferroelectric ceramic composed of 0.15PbTiO_3_–0.85PbSc_0.5_Ta_0.5_O_3_ (0.15PT–0.85PST), which exhibited excellent electrothermal effects at room temperature. By quenching, the electrothermal effect ($\Delta$ *T*_max_) and energy harvesting properties of the device were 1.574 K and 0.542 J/cm^3^, respectively. Then, for the first time, we proposed a self-powered temperature quantification control system with a rotary disc-shaped TENG. This device effectively harnessed wind and water energy, in addition to other types of energy. The system consisted of energy collecting cups, a rotating disc-shaped FEP–rabbit fur TENG, a circuit management module, and a ferroelectric ceramic chip array. Through the circuit management module, the system converted external wind energy into a high-voltage electric field at the two ends of the 0.15PT–0.85PST ceramic chip to fully stimulate the electrothermal effect. At a speed of 200 rpm, the temperature change in the insulated cup within 276 s was 0.49 K, and the volume of the insulated cup was 300 times greater than that of the 0.15PT–0.85PST ceramic chip. Compared with the results reported in previous work, the cooling and heating times were both reduced by 31%, and the temperature changes for both cooling and heating increased by 81%. Moreover, the heating and cooling temperatures of the device optimized on this basis were increased to 1.19 K and 0.93 K, respectively. The great improvement in the temperature variation performance confirmed the great potential of the device for commercialization. This research could serve as a reference for reducing energy consumption for cooling and heating, and it meets the international energy policies of carbon dioxide emission peaking and carbon neutrality.

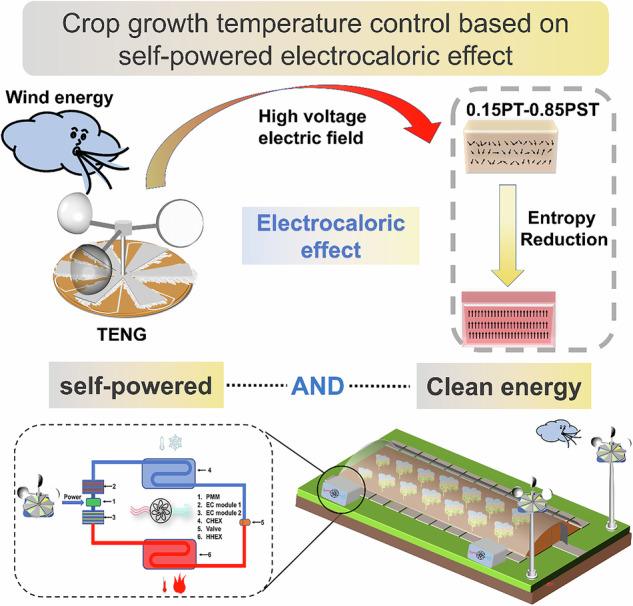

## Introduction

With the continuous development of society, heating and cooling processes consume more natural energy than before. Conventional cooling and heating processes are usually performed with compressors driven by electrical energy to control the Freon phase change characteristics; these processes often consume considerable amounts of power^1–3^. As the total amount of fossil fuel energy sources on Earth is decreasing, it is highly important to find a new self-powered technology^4–6^. Triboelectric nanogenerators, which were proposed by the academic Zhonglin Wang, can convert various forms of mechanical energy in the environment, such as wind^7–9^, wave^10–12^, water droplet^13–15^, and human body movement energy sources, into electrical energy^16–18^. With the advantages of a low production cost, a convenient transmission ability, and an excellent low-frequency energy harvesting effect, this technology has attracted extensive attention in many fields, such as sensors^19^, medicine^20^, agriculture^21^, and artificial intelligence^22^.

In addition, TENGs are self-powered technologies. These nanogenerators provide new contributions to research on functional materials (e.g., ferroelectric materials and thermoelectric materials) in temperature-variable devices. The Seebeck effects of thermoelectric materials and the electrothermal effects of ferroelectric materials are the foundational effects underlying the research and development of devices that can change temperatures. The Seebeck effect requires a stable high current (more than 1A) to serve as the basis; thus, this effect is not currently realized by TENGs. In contrast, ferroelectric materials only need a high electric field to induce stable electrothermal effects. Therefore, combining the high-voltage outputs of TENGs with the electrothermal effects is perfect for realizing self-powered cooling and heating processes.

Research on electrocaloric materials has been conducted for decades, and many scientific researchers around the world are committed to preparing electrocaloric materials with high EC performance characteristics^23,24^. The 350-nm PbZr_0.95_Ti_0.05_O_3_ thin film prepared by Mischenko and other researchers has a large electrocaloric effect at room temperature: Δ*T* = 12 K^25^. Peng and other researchers have prepared lead-free ferroelectric ceramics, which greatly reduce the damage to the environment^26^. In 2022, Li and other researchers first proposed the use of TENG and PSTT ceramics to realize self-powered electrocaloric refrigeration and heating^27^. In 2023, researchers, including Li, presented an electrothermal cooler with a maximum temperature of 20.9 K. The cooler reaches a maximum cooling power of 4.2 W under a moderate electric field of 10 V per micron^28^. On the one hand, due to the shortcomings of TENGs in the vertical separation mode, such as low voltage and slow charge accumulation, they cannot quickly create ceramic chips with adequate electric field strengths. On the other hand, the comprehensive performance characteristics of the ferroelectric ceramic materials used in the experiment, including energy capture and electrocaloric performance, still have room for improvement. Therefore, it is highly important to develop triboelectric nanogenerators with fast charge accumulation and high-voltage output abilities and ceramic materials with improved energy capture performance characteristics.

For the first time, this work presents a self-powered temperature-quantifiable control device (SPT 1.0) with a tunable structure. To accommodate the high electric field strength required for the ceramic chip to exert the electrothermal effect, an FEP–rabbit fur rotating turntable TENG is used. An ultrahigh open-circuit voltage of 6913 V at 240 rpm can be obtained by driving the continuous rotation of the turntable via wind energy. With only six 0.15PT–0.85PST ceramic chips, this experimental phenomenon is achieved beyond the 12 PSTT ceramic chips used in studies by Li and other researchers. A temperature change of 0.49 K is achieved in a space 300 times greater than the volume of the ceramic chips, which is an 81% improvement in the temperature change at room temperature. Moreover, the time required for cooling/heating is only 276 s, which is a 31% reduction. Furthermore, by increasing the number of ceramic pieces and adding cooling components, an optimized SPT of 2.0 increases the heating and cooling temperatures to 1.19 K and 0.93 K, respectively, confirming the commercialization of the device. Here, we only propose an application scenario for agricultural production. The system collects and converts energy from the variable natural environment into electricity, thereby controlling the temperature of the agricultural greenhouse. A solution is proposed for energy consumption due to cooling and heating during agricultural production.

## Experimental procedures

**Fig. 1:**
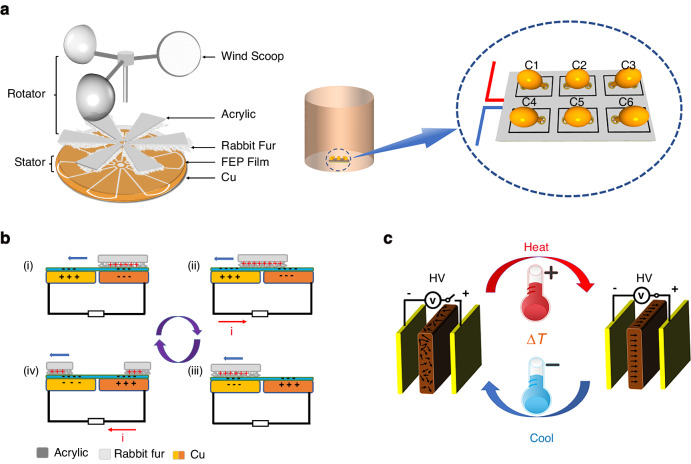
Schematic diagram of the self-powered system for temperature variation and the working principles of its components. **a** Schematic diagram of the FEP–rabbit fur TENG and ceramic chip array of a self-powered system that changes temperatures. **b** Schematic diagram showing the underlying mechanisms of the FEP–rabbit fur TENG. **c** Schematic diagram of the electrocaloric effect

Based on the electrocaloric effects of ferroelectric ceramics and the unique advantages of TENGs, we design a self-powered temperature-changing system composed mainly of an array of these components, as shown in Fig. 1a. The TENG is composed of a wind cup, bearing, stator and rotor. The wind cup is made of a light acrylic ball and is connected to the rotor, which is covered with rabbit fur through the bearing. The stator part is a customized disc coated with a copper electrode, and a layer of FEP film is pasted on the surface of the copper electrode. The working principle of rotary disc-shaped TENGs is shown in Fig. 1b. When electrode B is completely covered with rabbit fur, due to the contact electrification effect, some of the electrons on the rabbit fur can be transferred to the FEP film so that the friction FEP film exhibits negative charges. The electric potential of the upper surface of electrode B increases, and the negative charges on electrode A gather at electrode B through the external circuit for charge conservation, as shown in Fig. 1b(i). Then, because of external wind energy, the wind cup drives the rotation of the rotor. As rabbit fur gradually transfers from electrode B to electrode A, the potential on electrode B progressively decreases, and that on electrode A steadily increases. The positive charge gradually transfers to electrode B through the external circuit. Then, the negative charge gradually transfers to electrode A through the external circuit, forming a current between electrode A and electrode B, as shown in Fig. 1b(ii). When the rabbit fur is completely transferred to electrode A, the number of negative charges on electrode A peaks, and the current in the external circuit eventually disappears, as shown in Fig. 1b(iii). When rabbit fur transfers from electrode A to electrode B again, the potential on electrode A decreases steadily, and the potential on electrode B increases gradually. The negative charge slowly transfers to electrode B through the external circuit, and the positive charge progressively transfers to electrode A through the external circuit. This process forms a current from electrode B to electrode A, as shown in Fig. 1b(iv). When rabbit fur is completely transferred to electrode B, the number of negative charges on electrode B peaks, the current in the external circuit gradually disappears, and the rotary disc-shaped TENG returns to its original state. Following this cycle, under the effect of wind energy, the rotary table continuously outputs alternating current to the external circuit. Through circuit management, the charge transferred by the TENG regularly accumulates on the silver electrodes on both sides of the ceramic sheet, forming an electric field with continuously increasing intensity. This phenomenon results in the rearrangement of the electric domains in the ceramic sheet. The entropy in the ceramic sheet changes as the electric domains rearrange, resulting in heat exchange between the ceramic sheet and the external environment. This process is called the EC effect. The principle of the EC effect is detailed in Fig. S1 in the Electronic Supplementary Materials (ESM). By applying an electric field to the ceramic chip, heat is released to the surrounding environment for heating. When the charges on both sides of the ferroelectric ceramic are neutralized, due to the reduction in the electric field, the electric domain returns to the disordered state, and the ceramic material absorbs the surrounding heat energy for cooling. Therefore, by absorbing the wind energy in the surrounding environment, rotary disc-shaped TENGs can continuously provide electric energy for ceramic chips for heating or cooling.

## Results and discussion

**Fig. 2:**
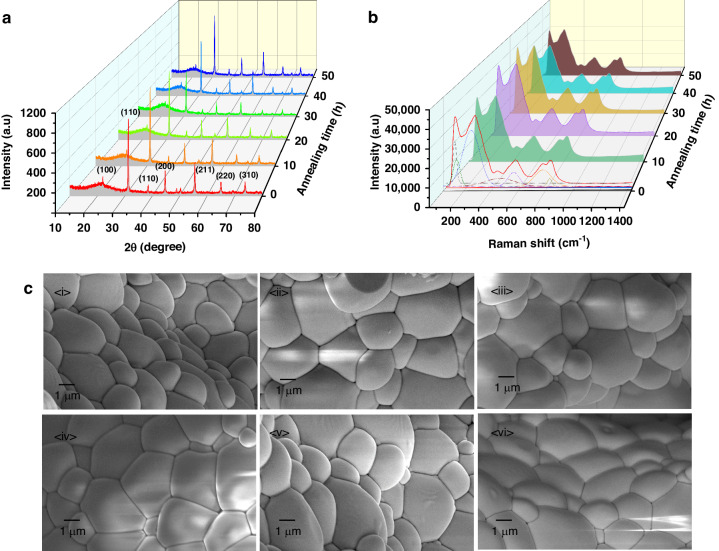
Crystal morphology and microstructure of 0.15PT-0.85PST. **a** XRD patterns of 0.15PT–0.85PST ceramics without quenching and with quenching times of 10–50 h. **b** Raman spectra of 0.15PT–0.85PST ceramics without quenching and with quenching times of 10–50 h. **c** SEM images of 0.15PT–0.85PST ceramics without quenching and with quenching times of 10–50 h

According to the phase diagram for Pb(Sc_0.5_Ta_0.5_)O_3_–PbTiO_3_(0.15PST–0.85PT), 0.15PT–0.85PST ferroelectric ceramics were prepared, and their EC properties were optimized by quenching for *xh* (*x* = 0, 10, 20, 30, 40, 50) (Fig. S2 in the ESM). The XRD patterns of the 0.15PT–0.85PST samples that have not been quenched or that have been quenched for 10–50 h are shown in Fig. 2a (Fig. S3 in the ESM). No obvious peak position variations are observed for the (100), (110), (111), (200), (211), (220), and (310) orientations. There are no other impurity peaks in the samples with or without quenching, which suggests that the quenching process does not cause significant changes in the crystal structures of the ceramics.

The Raman spectra in Fig. 2b (Fig. S4 in the ESM) show six typical peaks for the 0.15PT–0.85PST ceramics. The Raman spectra of each sample have six characteristic peaks that are fitted well. The peak near 100 cm^−1^ is mainly caused by the vibration of the a-site atom in the perovskite structure. The peak near 200 cm^−1^ is related not only to the vibration of the asymmetric O phonon in the orthogonal phase but also to the vibration in the rhombohedral phase. The peak near 300 cm^−1^ is mainly caused by the asymmetry of the phonon vibrations of TiO_2_, Sc_2_O_3_, and Ta_2_O_5_ in the tetragonal/orthogonal phases. The peak near 500 cm^−1^ is mainly caused by the symmetric tensile vibration of TiO_2_/Sc_2_O_3_ in the tetragonal twisted polar octahedral cluster. The peak near 700 cm^−1^ is mainly caused by the vibration of polar clusters, and its appearance is considered a relaxation feature. During quenching, no new peak or disappearance appears for the old band, indicating that the phase structure is relatively stable, which is consistent with the XRD results. In addition, from the SEM image shown in Fig. 2c, the grains of each PSTT sample are closely arranged without gaps, and the grain size distribution is shown in Fig. S5 in the ESM. The grain sizes of the PSTT ceramics increase linearly with increasing quenching time, mainly because small particles fuse into large particles with increasing quenching time. The above characterization results show that the prepared PSTT ceramics have good crystal quality, with good elemental distributions and dense microstructures.

**Fig. 3:**
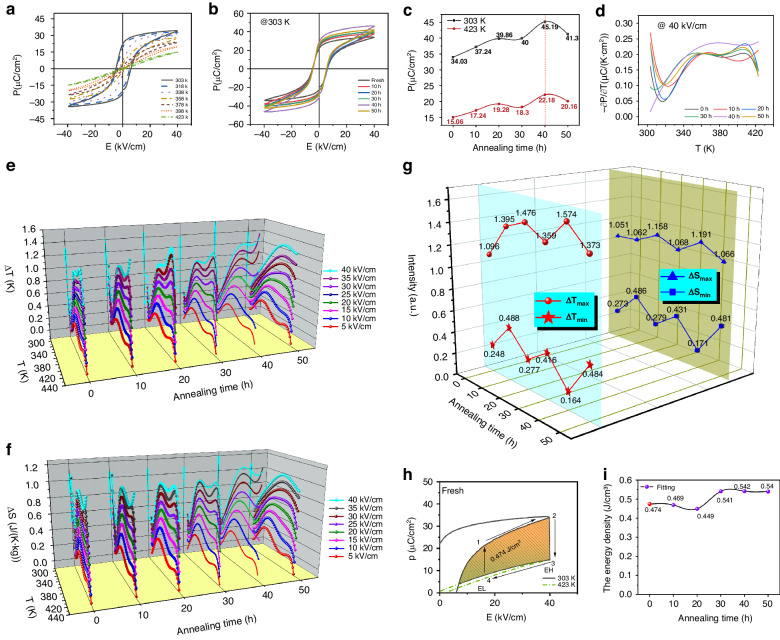
Effect of quenching process on ferroelectric properties of 0.15PT-0.85PST ceramics. **a** Hysteresis loops of 0.15PT–0.85PST ceramics without quenching and with quenching times of 10–50 h at 303 K. **b** Hysteresis loops of 0.15PT–0.85PST without quenching at 303–423 K. **c** Summary of the maximum polarization intensities of 0.15PT–0.85PST without quenching and with quenching times of 10–50 h at 303 K and 423 K. **d** Relationships between the pyroelectric coefficient (∂P/∂T) and temperature (*T*) values of 0.15PT–0.85PST ceramics without quenching and with quenching times of 10–50 h. **e**, **f** Relationship between the adiabatic temperature variation Δ*T* and the temperature (*T*). The relationship between the entropy variation Δ*S* and the temperature (*T*). **g** Statistical summary of the adiabatic temperature variation Δ*T* and the temperature. **h** Olsen cycle diagram of 0.15PT–0.85PST ceramics without quenching and with **i** quenching times of 10–50 h

At a test frequency of 1 Hz, the hysteresis loops of 0.15PT–0.85PST without quenching and with quenching times of 10–50 h at 303 K are shown in Fig. 3a (Fig. S6 in the ESM). The shapes of the hysteresis loops of all the 0.15PT–0.85PST ceramics are wide and sharp, with relatively large remaining polarization intensities. The maximum polarization occurs when the electric field intensity peaks, and no leakage occurs, which proves that the material is uniform and dense and that the crystallinity is high. At temperatures of 303–423 K, the hysteresis loop of 0.15PT–0.85PST without quenching is shown in Fig. 3b, and those of the quenched 0.15PT–0.85PST samples are shown in Fig. S7 in the ESM. The hysteresis loop of 0.15PT–0.85PST without quenching is wide and sharp at 303 K, and the maximum polarization intensity reaches 34.4 μC/cm^2^ at 303 K. With increasing test temperature, 0.15PT–0.85PST shows obvious temperature sensitivity. Specifically, the hysteresis loop is gradually narrowed, the coercivity field is significantly reduced, and the maximum polarization intensity is significantly reduced. When the temperature reaches 423 K, the maximum polarization intensity is only 14.9 μC/cm^2^. At either 303 K or 423 K, 0.15PT–0.85PST with a quenching time of 40 h shows the maximum polarization, as shown in Fig. 3c. Moreover, the polarization of 0.15PT–0.85PST at 303 K is more than twice that at 423 K, which proves that the electrocaloric effect of 0.15PT–0.85PST has great potential at 303 K. The relationship between the pyroelectric coefficient (partial P/partial T) and temperature (T) is shown in Fig. 3d. The best pyroelectric performance characteristics of PST that are quenched for 10–50 h and that are not quenched are both demonstrated below 325 K. Moreover, at 303 K after quenching for 40 h, 0.15PT–0.85PST shows the maximum pyroelectric coefficient.

Under approximately reversible adiabatic heat, Maxwell’s equation (*∂P*/*∂T*)_E_ = (*∂S*/*∂E*)_T_ is considered valid. The effects of EC on these ceramic samples are determined as follows:

1 $$\Delta T=-\frac{1}{\rho }{\int }_{{E}_{1}}^{{E}_{2}}\left(\frac{T}{C}\right)\left(\frac{{\partial }_{P}}{{\partial }_{T}}\right){EdE}$$

2 $$\Delta S=-\frac{1}{\rho }{\int }_{{E}_{1}}^{{E}_{2}}\left(\frac{{\partial }_{P}}{{\partial }_{T}}\right){EdE}$$

where *P* is the maximum polarization intensity in the corresponding electric field, *T* is the temperature, and *E*_1_ and *E*_2_ are the initial electric field and the final electric field, respectively. The adiabatic temperature variation Δ*T* and entropy variation Δ*S* of the unquenched and quenched PSTT samples with temperature (*T*) are shown in Fig. 3e and Fig. 3f, respectively (Figs. S8 and S9 in the ESM). With increasing temperature, the adiabatic temperature variation Δ*T* and entropy variation Δ*S* first decrease, then increase and finally decrease with increasing temperature (*T*). With increasing electric field intensity, the adiabatic temperature variation Δ*T* and entropy variation *ΔS* gradually increase, which indicates that the electrocaloric effect of the 0.15PT–0.85PST ceramic is extremely sensitive to temperature, as shown in Fig. 3g. Under an electric field intensity of 40 kV/cm, the impact of quenching time on the adiabatic temperature variation Δ*T* is large in terms of their relationship. The largest Δ*T* occurs at a quenching time of 40 h, and Δ*T*_max_ is 1.574 K. The smallest Δ*T* occurs at a quenching time of 40 h, and Δ*T*_min_ is 0.164 K. The entropy variation Δ*S* with quenching time is similar to the adiabatic temperature variation Δ*T*. Therefore, 0.15PT–0.85PST ceramics with quenching times of 40 h have excellent electrocaloric properties, and the electrocaloric properties are most sensitive to temperature.

Figure 3h shows the Olsen cycle diagram of 0.15PT–0.85PST without quenching. The energy density captured by the closed cycle is equal to the area surrounded by cycle 1 → 2 → 3 → 4. The energy capture formula is as follows:

3 $$W=\oint {EdP}$$

where *E* is the electric field intensity, and *P* is the polarization strength. The energy capture of the 0.15PT–0.85PST sample without quenching is 0.474 J/cm^3^. The cycle diagrams of the quenched PSTT ceramics are shown in Fig. S10 in the ESM. Figure 3i presents a summary of the energy densities of the PSTT ceramics. Similarly, 0.15PT–0.85PST ceramics quenched for 40 h have the best energy capture capacities, with a maximum value of 0.542 J/cm^3^. In summary, the PSTT ceramics quenched for 40 h have the best EC performance and are selected as materials for the follow-up experiment.

**Fig. 4:**
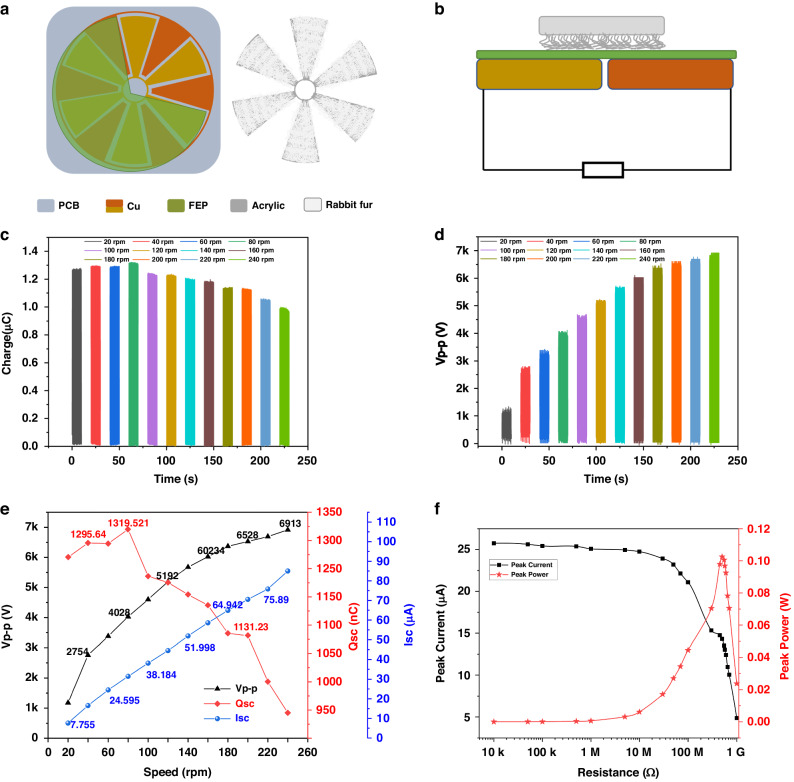
The structural and electrical properties of the rotary disc-shaped TENG. **a** Exploded view of the rotary disc-shaped TENG. **b** Side view of rotary disc-shaped TENG. **c** Transferred charge. **d** Peak-to-peak voltage. **e** Trend of the output versus rotation speed in the TENG. **f** Trend of the power versus the external load resistance

Considering the impact of the 0.15PT–0.85PST ceramic electrocaloric effect on high-voltage, a rotary disc-shaped FEP–rabbit fur TENG is made. The disassembly diagram is shown in Fig. 4a. The stator base is made of a PCB board, on which a circular copper electrode with a radius of 13 cm is plated. The circular electrode is composed of two groups of electrodes, each of which is made of six sector electrodes in series. The electrode is covered with an FEP film layer. The rotor is made of an acrylic plate cut with a laser. The size of the acrylic plate matches the size of the copper electrode, and 3 mm of rabbit fur is uniformly stuck on the acrylic board. The distance between the stator and the rotor is controlled at approximately 2.5 mm to ensure that the rabbit fur and the copper electrode are fully contacted without excessive resistance. To explore the effect of rotating speed on TENG output, the rotating speed of the rotary table is controlled by the linear motor in Fig. 4b to obtain different TENG outputs. Figure 4c, Fig. 4d and Fig. S11 (ESM show the transferred charge, open-circuit voltage and short-circuit current of the TENG, respectively. Fig. S12 in the ESM shows a clear output curve. Figure 4e shows the broken line diagram of the changes in the TENG output with the rotation speed. Overall, the transfer charge decreases with increasing rotation speed, which may be caused by insufficient contact between the rabbit fur and the FEP film due to the increase in rotation speed. However, the open-circuit voltage increases with increasing speed—only 1177 V at 20 rpm and nearly 6913 V at 240 rpm. The trend of the short-circuit current is similar to that of the voltage, and the current increases with increasing speed from an initial value of 7.755–85 μA. Therefore, the rotary disc-shaped FEP–rabbit fur TENG has excellent output capability, and its unique high-voltage output capability is particularly suitable for stimulating the electrocaloric effects of ferroelectric materials. Finally, the variation in the TENG peak output power with respect to the external load resistance is tested, and the results are shown in Fig. 4f.

4 $$P={I}_{t}^{2}R$$

where *P* is the peak power, *I*_*t*_ is the peak current, and *R* is the external resistance. The TENG has a maximum peak output power of 0.19 W at a resistance of 600 MΩ.

**Fig. 5:**
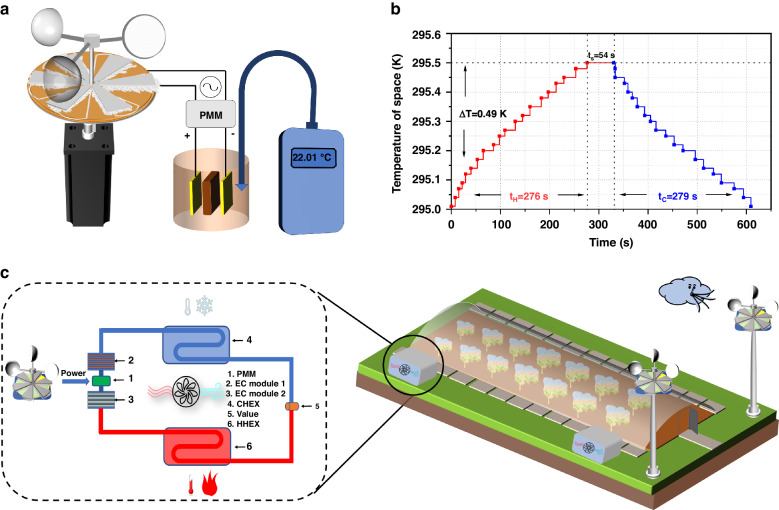
Schematic diagram of the self-powered temperature-changing test system (SPT 1.0) and its application to crop cultivation in agricultural greenhouses. **a** Schematic diagram of the self-powered temperature-changing test system (SPT 1.0). **b** Temperature variations in the vacuum cup when the rotary machine began to operate. **c** Schematic diagram of the mechanisms by which the self-powered temperature-changing system controls the temperature in a greenhouse

By leveraging the electrothermal effects of 0.15PT–0.85PST and the energy harvesting properties of the TENG, we develop a wind-powered, self-sustaining variable temperature testing system, which is designated SPT 1.0, as shown in Fig. 5a. The SPT 1.0 system is composed of a TENG, a management circuit (Fig. S12 in the ESM), an array of 0.15PT–0.85PST ceramic chips (comprising six units), a vacuum-sealed cup, and a high-precision thermometer. While the TENG remains stationary, the temperature within the vacuum cup is stable for 30 min. Upon activation, the TENG efficiently converts mechanical energy into electrical energy, creating a high-voltage electric field across the ends of the 0.15PT–0.85PST ceramic chips within the vacuum cup through circuit control. The electric field induces heating in the 0.15PT–0.85PST array due to its electrothermal effect. Once the TENG stops rotating, the accumulated charges at the ceramic chip ends are gradually discharged. Simultaneously, the temperature changes within the vacuum cup are monitored in real-time using a high-precision thermometer. The temperature change profile throughout the process after the rotary motor is initiated is shown in Fig. 5b. At 276 s, a temperature increase of 0.49 K is recorded within the vacuum cup, which remains constant at its peak value. After the electric field is removed from both ends of the 0.15PT–0.85PST ceramic sheet, the temperature rapidly decreases, returning to its initial state within 279 s (Video 1). Compared to previously published research^27^, the cooling and heating times are reduced by 31%, and the magnitude of temperature change that can be achieved through both cooling and heating can be increased by 81%. This improvement is primarily attributed to the FEP–rabbit fur rotating disc-shaped TENG, which exhibits excellent electrical performance, and 0.15PT–0.85PST, which demonstrates enhanced electrothermal performance. Furthermore, to enhance the thermal performance of the device and achieve subambient cooling effects, we refined the device, culminating in a new model called SPT 2.0 (Fig. S14). On the one hand, we have integrated controllable heat dissipation components into the original system to effectively achieve cooling below room temperature. On the other hand, we have increased the number of ceramic plates to significantly amplify the cooling temperature change capability of the device. We conduct tests on the cooling effect of the new device (Video 2). The operational process of SPT 2.0 differs from that of SPT 1.0. After undergoing the same initial heating process, SPT 2.0 introduces an additional heat conduction phase before the cooling process, during which the temperature inside the cup first decreases to a value near room temperature. Subsequently, the cooling process is initiated, and the temperature inside the cup decreases for a second time due to an increase in system entropy, resulting in subambient cooling (further details on the principles and processes can be found in Fig. S15). The results indicate that the temperature change of SPT 2.0 becomes substantially better than that of SPT 1.0. In the first phase of the heating process, SPT 2.0 achieves a temperature change of 1.19 K (from 296.48 K to 297.67 K), and in the third phase of the cooling process, SPT 2.0 achieves a cooling effect of 0.93 K (from 296.58 K to 295.65 K). Notably, SPT 2.0 successfully reduces the temperature inside the cup to 0.83 K below room temperature, demonstrating that an increased number of ceramic plates increases the change in cooling temperature. The outdoor environment abounds with wind energy, and rotating disc-shaped TENGs have natural advantages in harvesting low-frequency and disordered mechanical energy. Considering the significant impact of temperature on crop growth, we propose an agricultural production application scenario, as illustrated in Fig. 5c. The system harnesses environmental wind energy and converts it into electricity, which then regulates the temperature necessary for crop cultivation in agricultural greenhouses. This approach is undoubtedly a more environmentally friendly and cost-effective solution than other approaches.

## Conclusions

In summary, 0.15PbTiO_3_–0.85PbSc_0.5_Ta_0.5_O_3_ (0.15PT–0.85PST) ferroelectric ceramics with excellent electrothermal effects at room temperature were prepared. The electrothermal effect (Δ*T*_max_) and energy trapping performance characteristics of the ceramics were further optimized to 1.574 K and 0.542 J/cm^3^, respectively, by a quenching process. Moreover, a rotating TENG composed of FEP and rabbit fur with a maximum open-circuit voltage of 6913 V, a maximum short-circuit current of 85 μA, and a maximum transfer charge of 1.3 mC was prepared. Through a circuit management system, a self-actuated temperature quantization control device based on an FEP–rabbit fur TENG was successfully designed for the first time. A temperature variation of 0.49 K was achieved in a space 300 times greater than the volume of the ceramic chip. Compared with the state-of-the-art device, this device increased the degree to which the temperature was changed at room temperature by 81%. Moreover, the time required was only 276 s, and the cooling/heating times were both reduced by 31%. The further optimization of SPT 2.0 with relatively great heating (1.19 K) and cooling (0.93 K) temperature variations demonstrated the potential of the device for commercialization. Finally, we proposed one application scenario in agricultural production that could effectively reduce the energy consumption for cooling or heating in greenhouse agricultural production. This study could provide a reference for the research and development of cooling and heating devices at room temperature and respond to the international energy policy of carbon dioxide emission peaking and carbon neutrality.

## Experimental details

### Preparation of 0.85PST–0.15PT ceramics by the solid-phase sintering method

PbO_2_ (purity ≥97%), Ta_2_O_5_ (purity ≥99.99%), Sc_2_O_3_ (purity ≥99.9%), and TiO_2_ (purity ≥99%) are selected as the raw materials. The raw materials are milled for 12 h by ball milling. After drying, the samples are pressed into cylinders with customized molds and calcined for more than 2 h in an 1173 K environment. The calcined powder is ball milled again for 12 h and passed through a fine sieve. The resulting powder is pressed into sheets, and the ceramic sheets are compacted at 300 MPa. Finally, the ceramics are first fired at 1523 K and then quenched at 1373 K for 10–60 h. The resulting ceramic sheet is polished to 0.59 mm, and both ends are coated with silver electrodes (Fig. S13 in the ESM).

### Preparation of rotary TENG

A PCB board coated with a Cu film is used as the TENG stator. There are 6 pairs of electrodes on the stator, and a 0.1-mm-thick FEP is covered on the electrodes. Friction materials include 2-mm-thick rabbit hair and 0.1-mm-thick FEP. The stators are cut from acrylic sheets with rabbit hair attached to them. The bearing is made of acrylic rods to connect the stator and rotor. The wind cup is made of acrylic balls cut into semicircles and connected to acrylic rods.

### Characterization of the 0.85PST–0.15PT ceramics

The microstructure of 0.85PST–0.15PT is measured by scanning electron microscopy (Pro X, Phenom, Eindhoven, Netherlands). The XRD patterns of the ceramics are measured by a PANalytical X’Pert PRO instrument under Cu Kα radiation (*λ* = 15.406 nm). The polarization as a function of the electric field is measured by a ferroelectric analyzer (TF Analyzer 2000, Aix ACCT, Aachen, Germany).

### Electrical performance testing

The rotating motion of a TENG under different wind speeds is simulated via transfer. The voltage is measured by a high-voltage probe (TEKTRONIX P6015A) and an oscilloscope (TEKTRONIX MDO3014). The current is measured by an electrometer (Keithley 6514).

### Electronic supplementary material

Supplementary material (Additional figures for the principle of the electrocaloric effect include the following four stages; phase diagram for the Pb(Sc_0.5_Ta_0.5_)O_3_–PbTiO_3_(PST-PT); the XRD spectra of the 0.15PT–0.85PST ceramics; the Raman spectra of the 0.15PT–0.85PST ceramics; the particle size distribution of the 0.15PT–0.85PST ceramics; the P–E hysteresis loops of the PSTT ceramics with quenching; the relationship between the adiabatic temperature variation Δ*T* and the temperature (T) with quenching and the relationship between the entropy variation Δ*S* and the temperature (T) with quenching; the Olsen cycle diagram of pyroelectric energy harvesting of the PSTT ceramics with quenching; the short-circuit current, open-circuit voltage, and enlarged view of the transferred charge of the TENG at different rotational speeds were measured at 200 rpm; circuit simulation of the thermostat; diameter and thickness of the 0.15PT–0.85PST ceramic sheet).

## Supplementary information

supporting information

video 1

video 2
