# Supplementary material for: Self-powered temperature-changing system driven by wind energy
Source: Microsyst Nanoeng. 2024 Sep 26;10:135. doi: 10.1038/s41378-024-00741-1 (PMC11427466; doi:10.1038/s41378-024-00741-1)
Supplement: Supplementary file 1 — supporting information [file 41378_2024_741_MOESM1_ESM.docx]

Electronic Supplementary Material

**Self-powered temperature-changing system driven by wind energy**

Jiayu Li^1,5^, Boxun Liu^1,5^, Mingyang Li^2,4^, Yahui Li^6^, Wangyang Ding^1^, Guanlin Liu^1^, Jun Luo^3^, Nan Chen^1,5^, Lingyu Wan^1,5,*^ , Wenjuan Wei^2,*^

^1^ Center on Nanoenergy Research, Guangxi Colleges and Universities Key Laboratory of Blue Energy and Systems Integration, carbon peak and neutrality science and technology development institute, School of Physical Science and Technology, Guangxi University, Nanning 530004, China

^2^ Department of Chemistry and the Tsinghua Center for Frontier Polymer Research, Tsinghua University, Beijing, 100084 P. R. China

^3^ State Key Laboratory of Featured Metal Materials and Life-cycle Safety for Composite Structures, MOE Key Laboratory of New Processing Technology for Nonferrous Metals and Materials, and School of Resources, Environment and Materials, Guangxi University, Nanning 530004, China.

^4^ State Key Laboratory of Pollution Control and Resource Reuse, School of the Environment, Nanjing University, Nanjing 210023, China

^5^ State Key Laboratory of Featured Metal Materials and Life-cycle Safety for Composite Structures, Nanning 530004, China

^6^ Department of Micro/Nano Electronics, School of Electronic Information and Electrical Engineering, Shanghai Jiao Tong University, Shanghai 200240, PR China


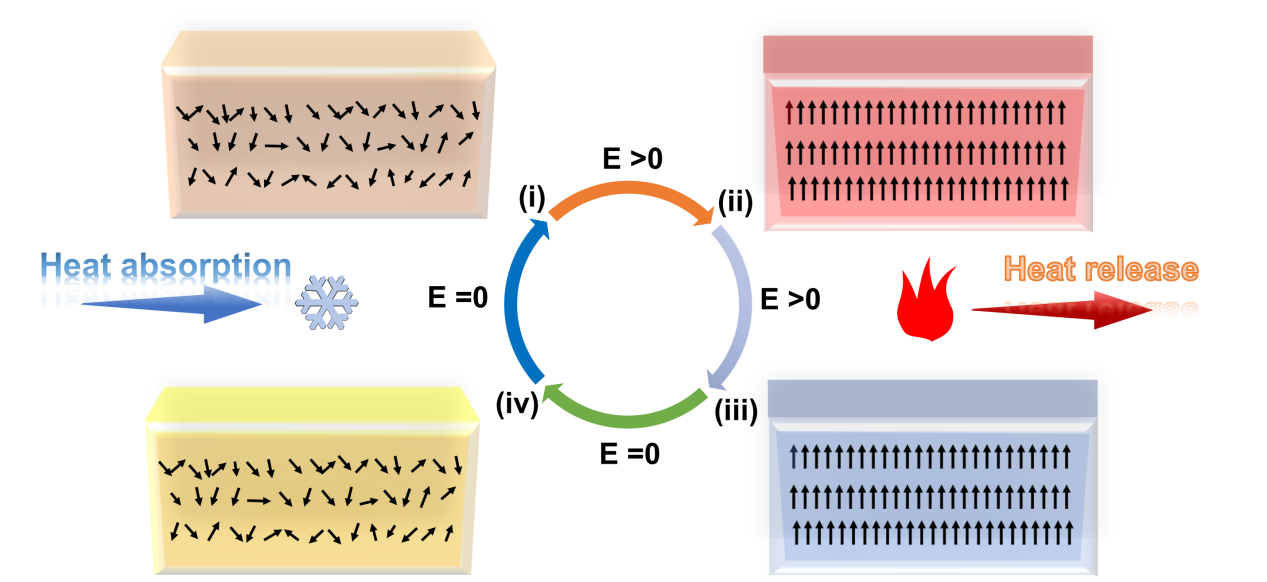


Fig. S1. The principle of the electrocaloric effect is demonstrated through the following four stages: (a) In the first stage, no electric field is applied to the EC material and the material temperature is room temperature. In the initial stage, the internal dipole of matter is disordered and the entropy is large. (b) In the second stage, the material is polarized under the adiabatic condition, that is, the external electric field acts on the EC material. Then the arrangement of internal dipoles gradually becomes orderly. The entropy of the material gradually decreases and the temperature gradually increases. This material will release heat to the outside world. (c) In the third stage, keeping the applied electric field unchanged, EC materials exchange heat with the external environment. The final material temperature is consistent with the ambient temperature. (d) In the fourth stage of removing the electric field under the adiabatic condition, the dipole inside the EC material changes from order to disorder. The entropy increases gradually and the material temperature decreases gradually. This material absorbs heat from the outside.


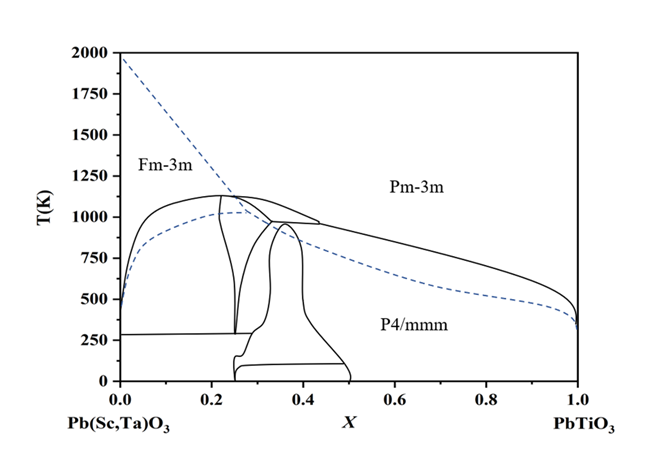


Fig. S2. Phase diagram for the Pb(Sc0.5Ta0.5)O3-PbTiO3 (PST-PT)


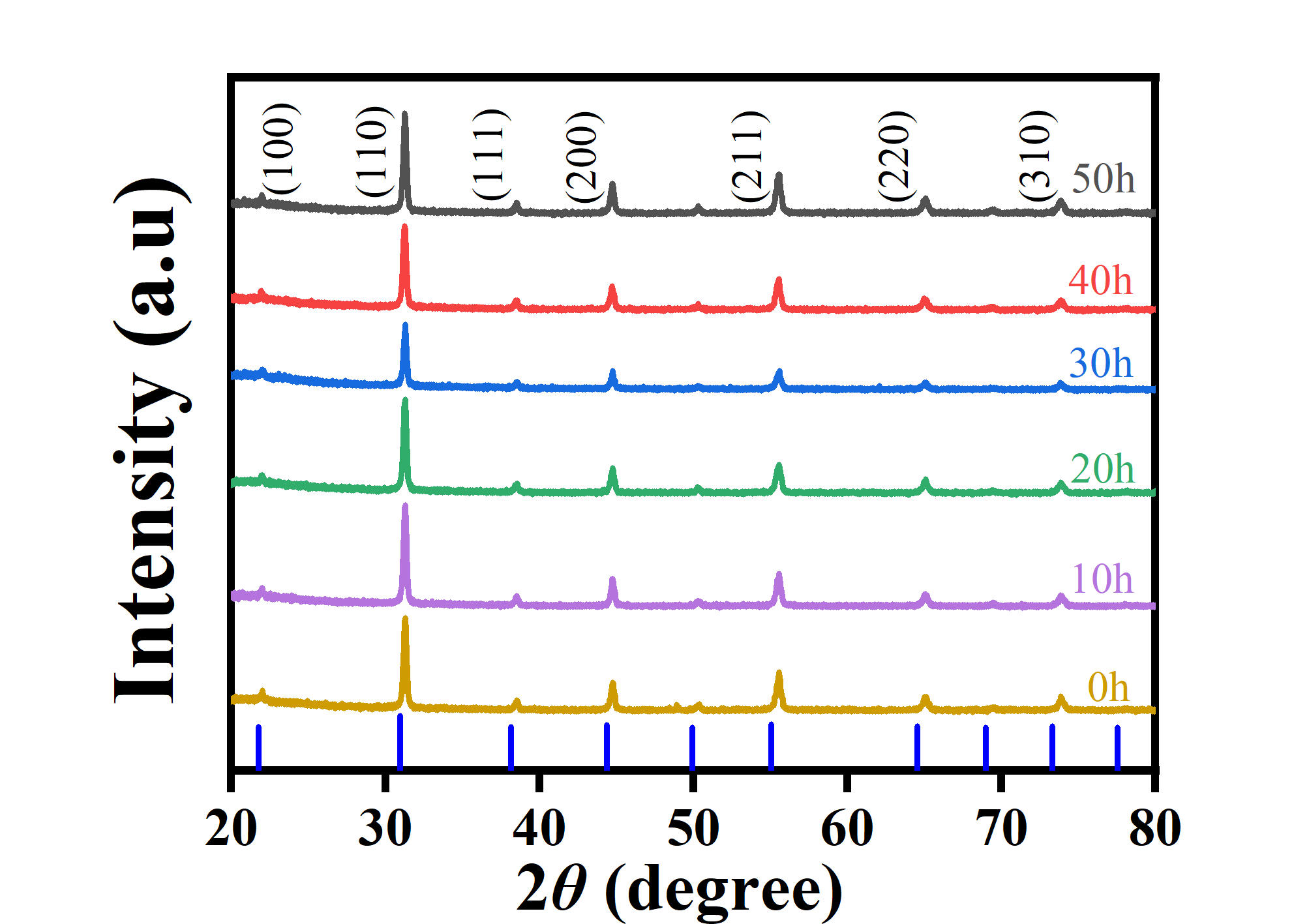


Fig. S3. The XRD spectra of 0.15PT-0.85PST ceramics without quenching and with quenching time of 10-50 hours


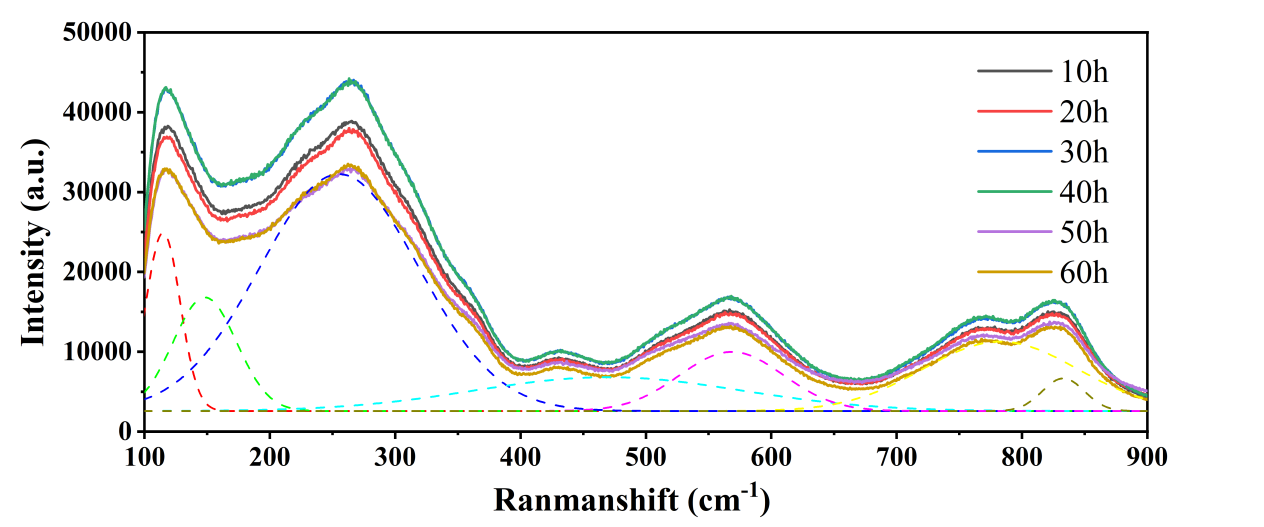


Fig. S4. The Raman spectra of 0.15PT-0.85PST ceramics without quenching and with quenching time of 10-50 hours


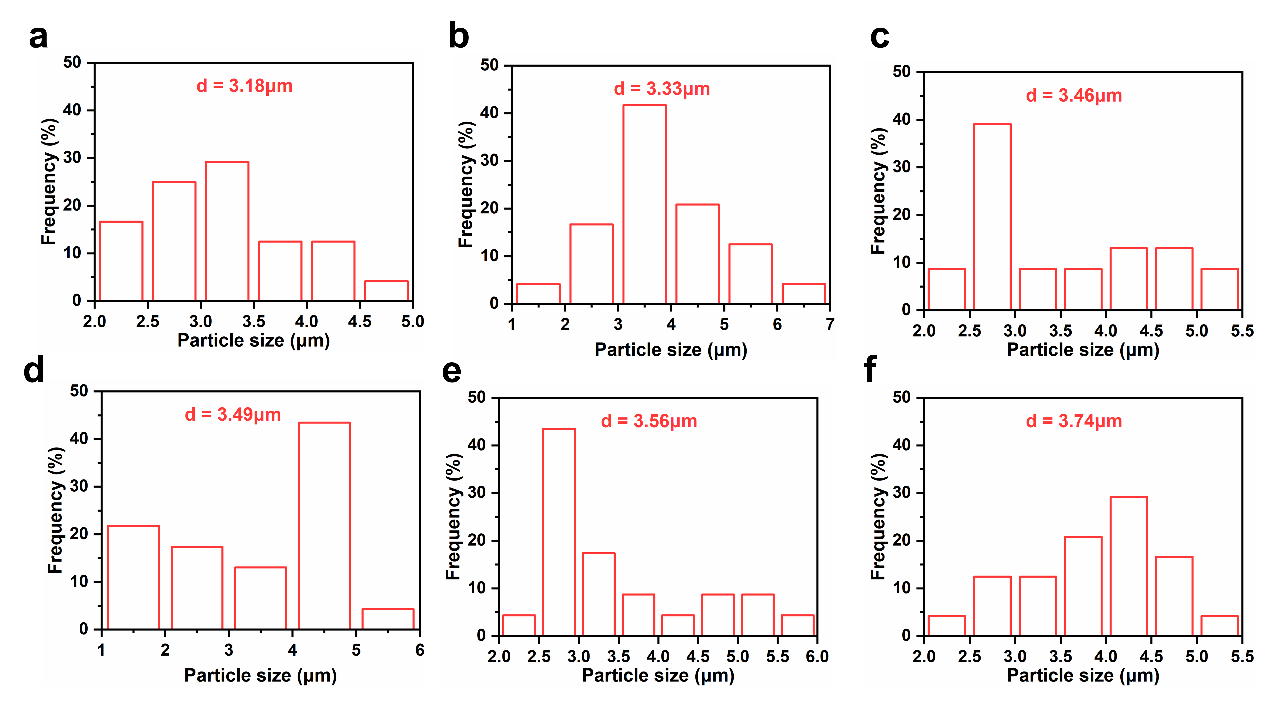


Fig, S5. Particle size distribution of 0.15PT-0.85PST ceramics (a) unquenched and quenched for (b)10h, (c)20h, (d)30h, (e)40h, (f)50h.


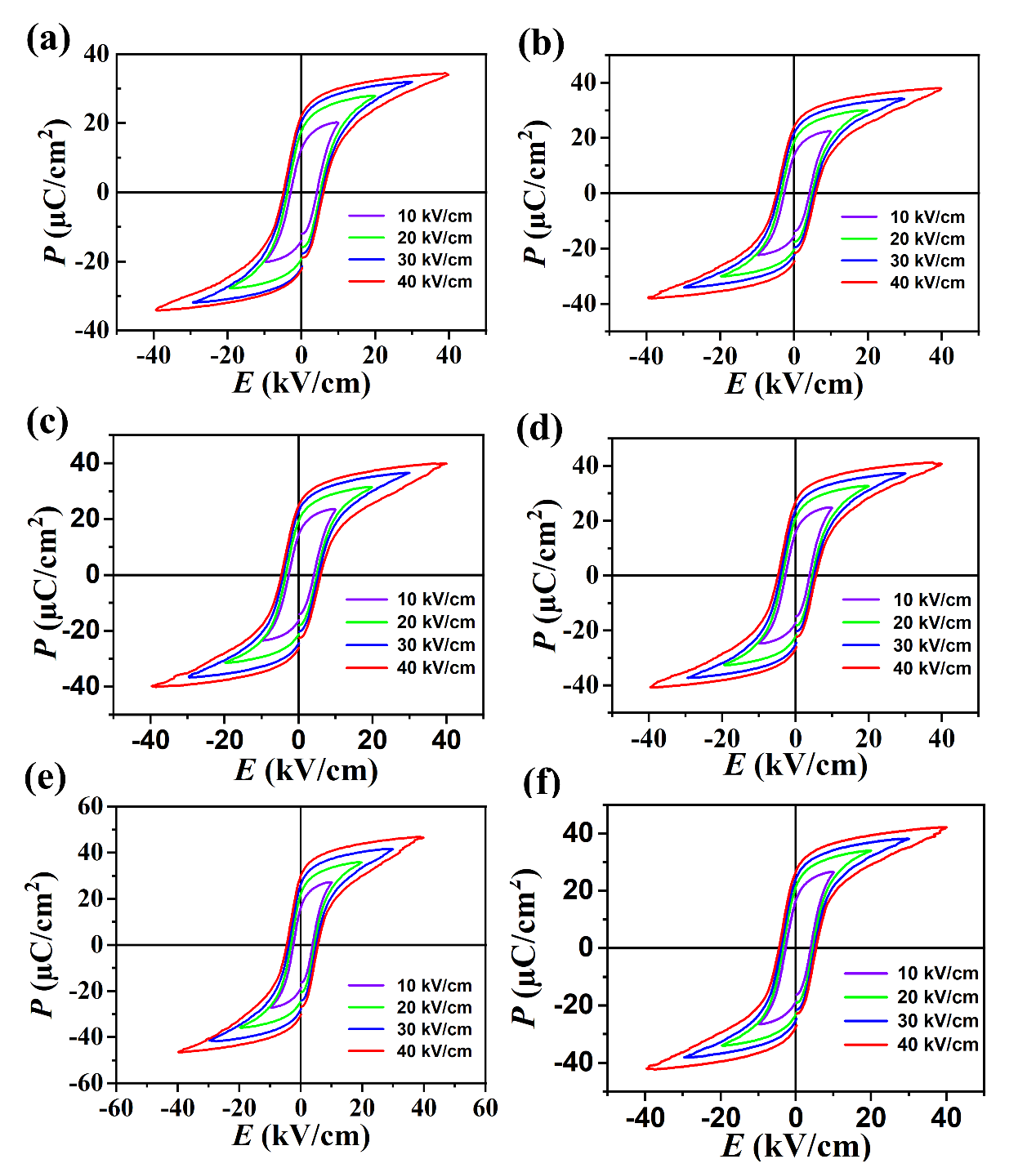


Fig. S6. P-E hysteresis loops of 0.15PT-0.85PST ceramics for different quenching times at selected electric fields. (a) x = 10 h, (b) x = 20 h, (c) x = 30 h, (d) x = 40 h, (e) x = 50 h, and (f) x = 60 h.


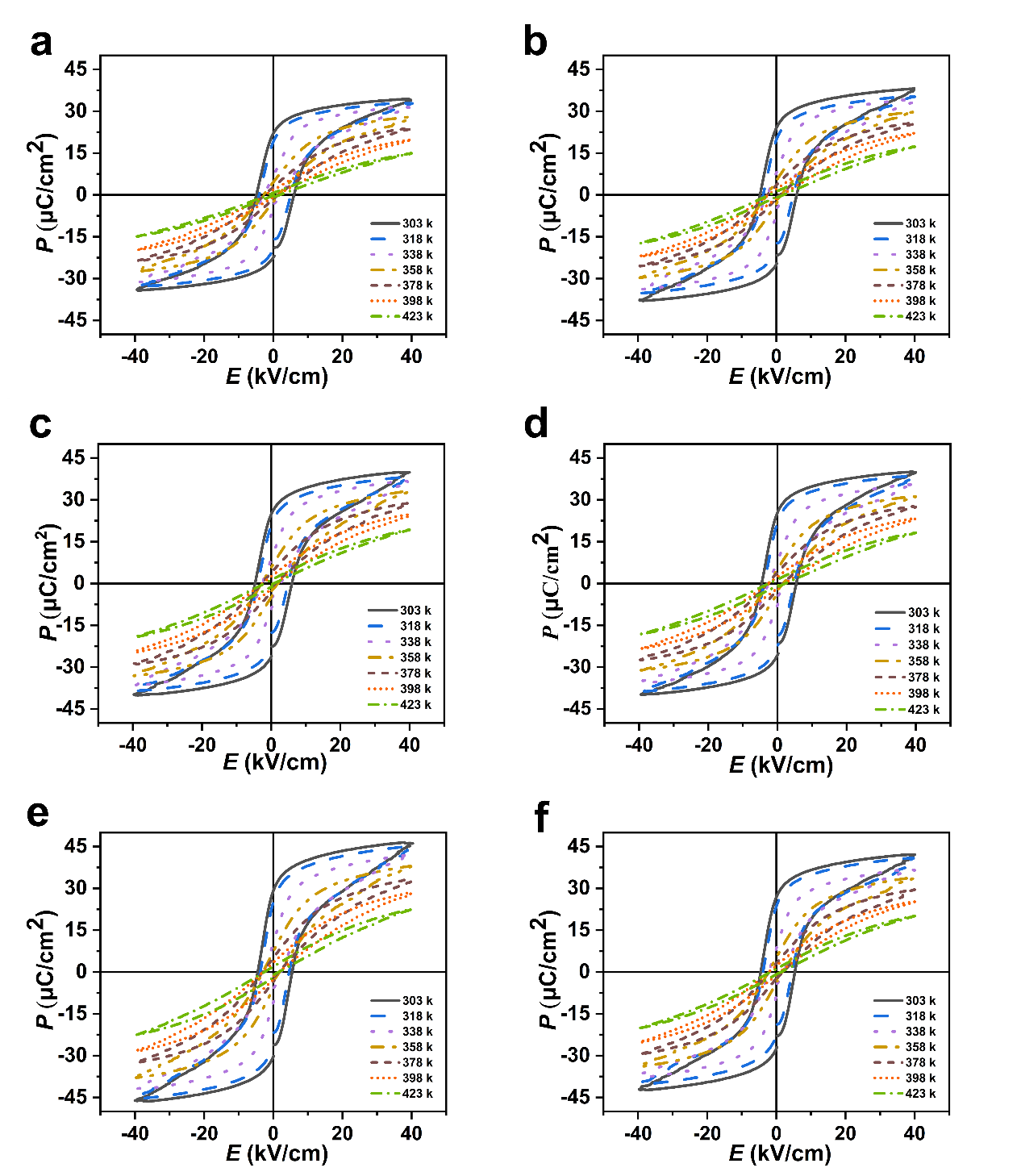


Fig. S7. P-E hysteresis loops of 0.15PT-0.85PST ceramics for different quenching times (a) x = 10 h, (b) x = 20 h, (c) x = 30 h, (d) x = 40 h, (e) x = 50 h, and (f) x = 60 h


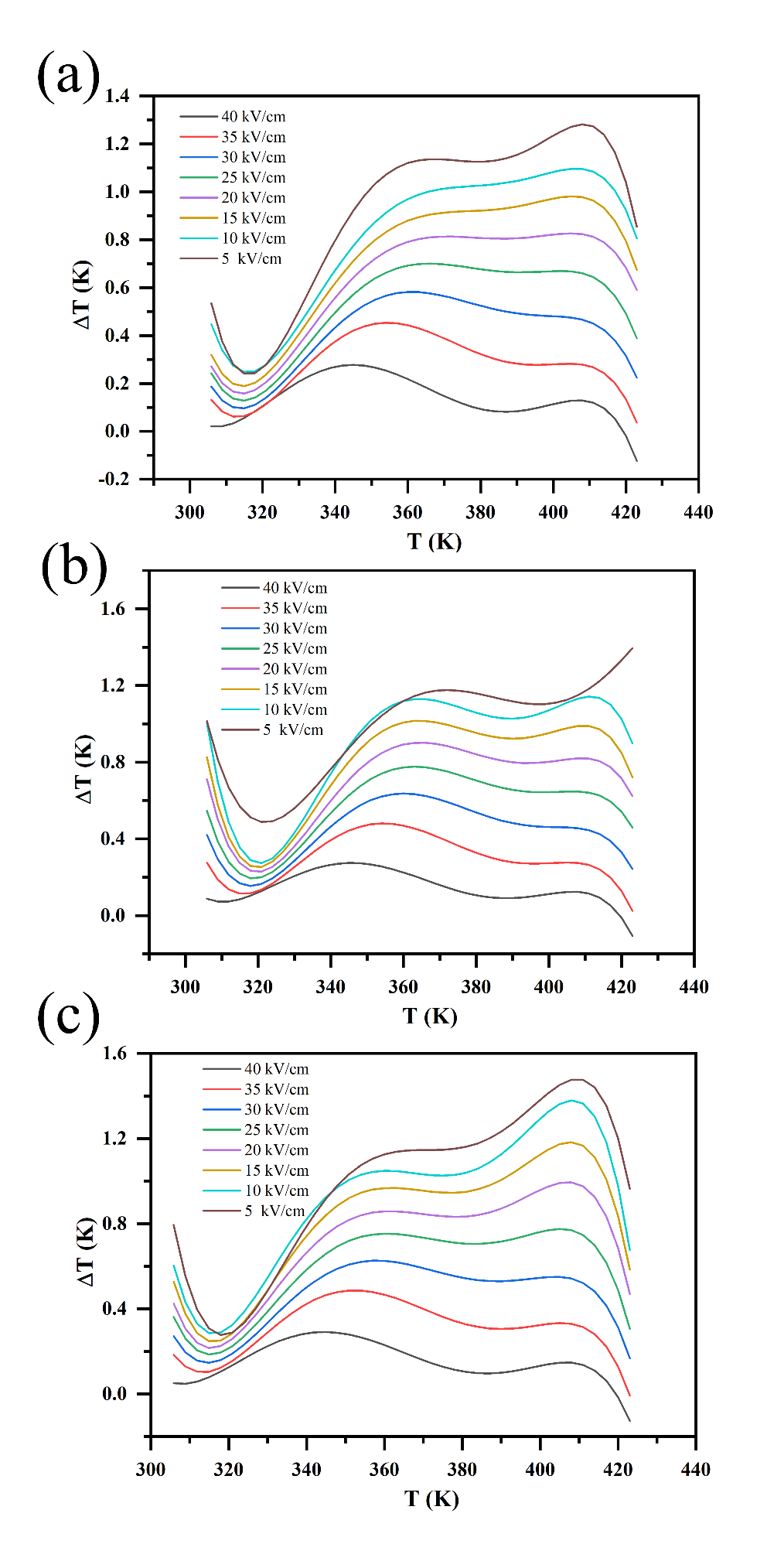


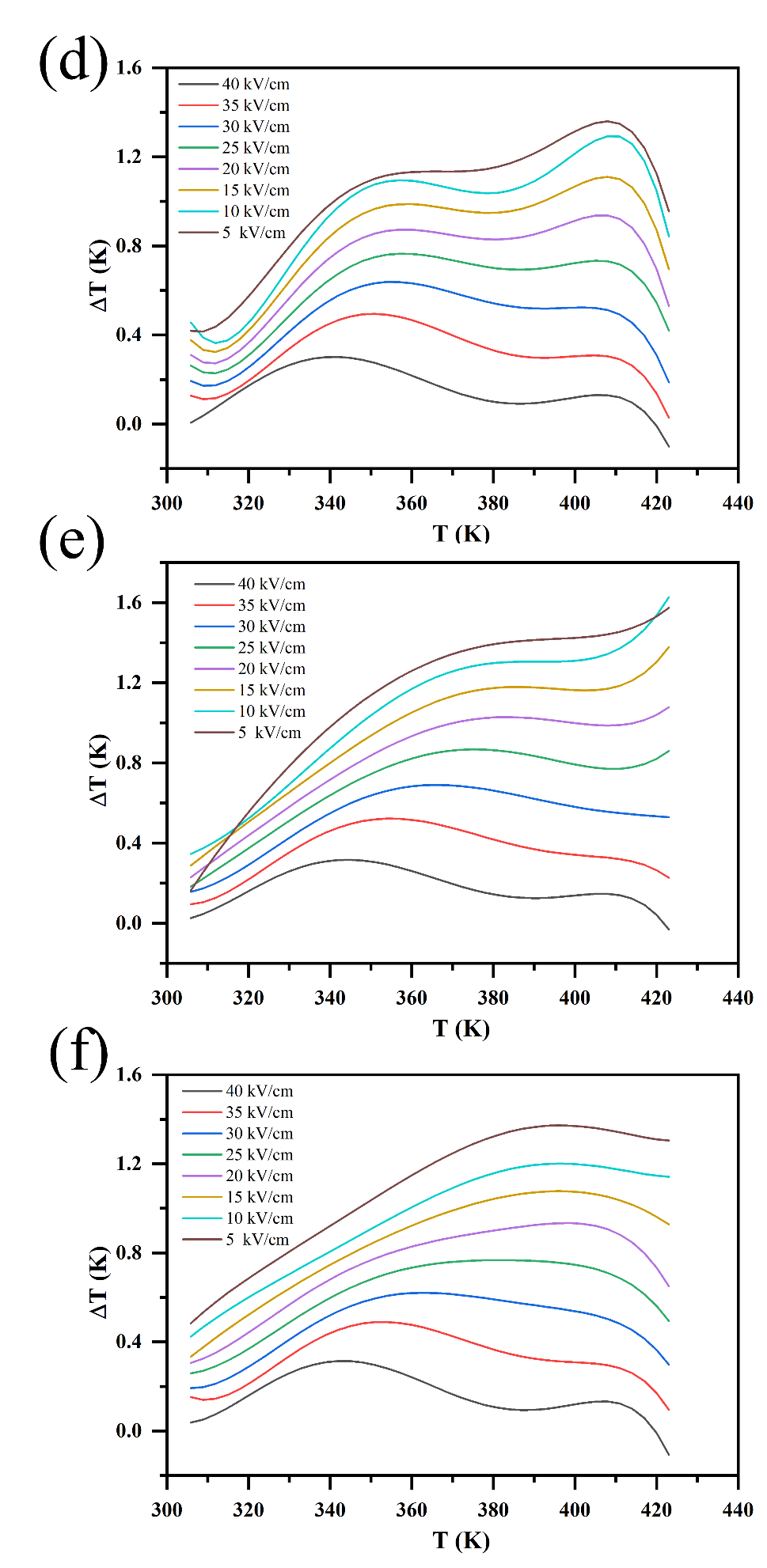


Fig. S8. The relationship between the adiabatic temperature variation ΔT and the temperature (T) for different quenching times (a) x = 0 h, (b) x = 10 h, (c) x = 20 h, (d) x = 30 h, (e) x = 40 h, and (f) x = 50 h.


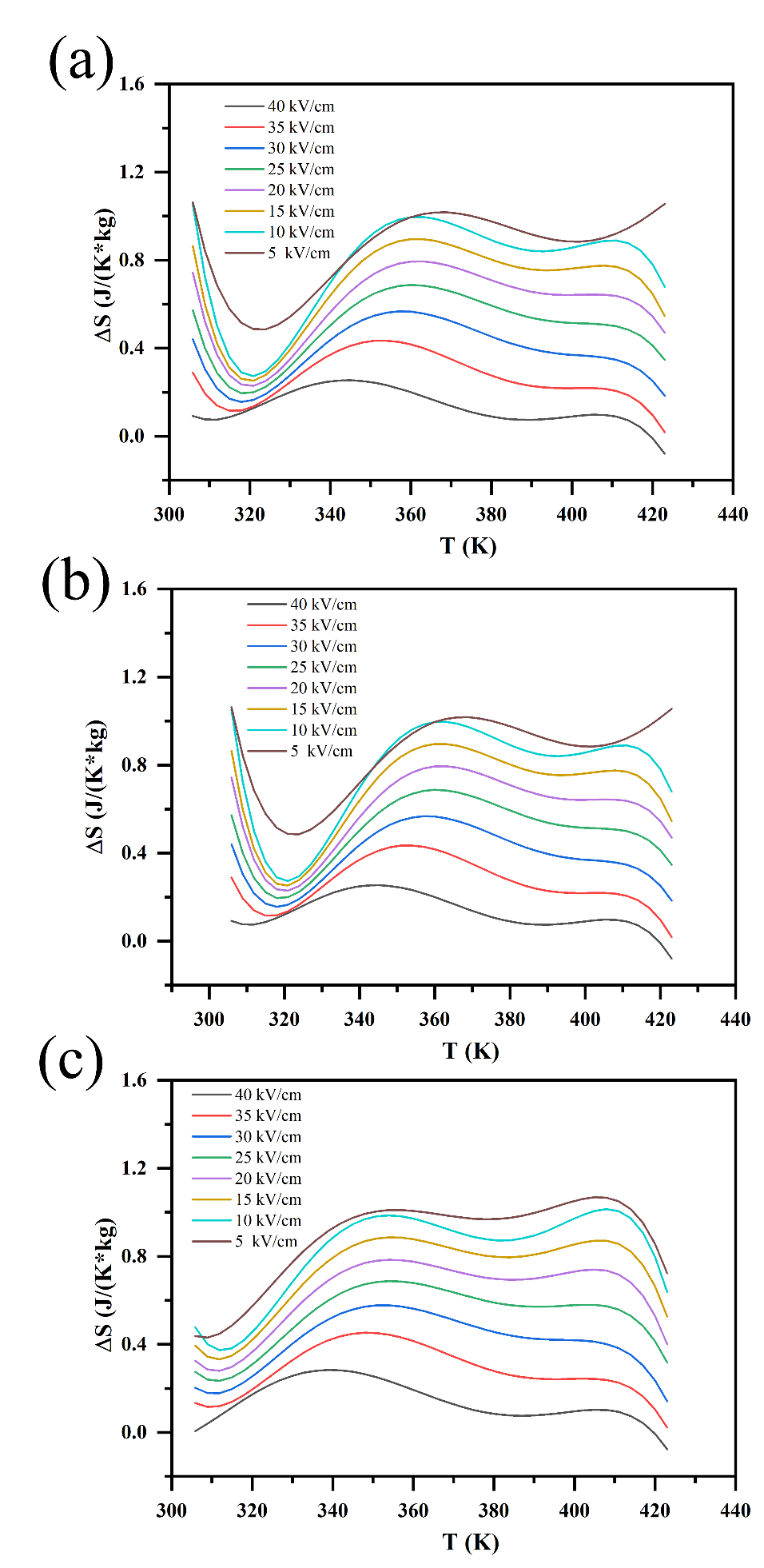


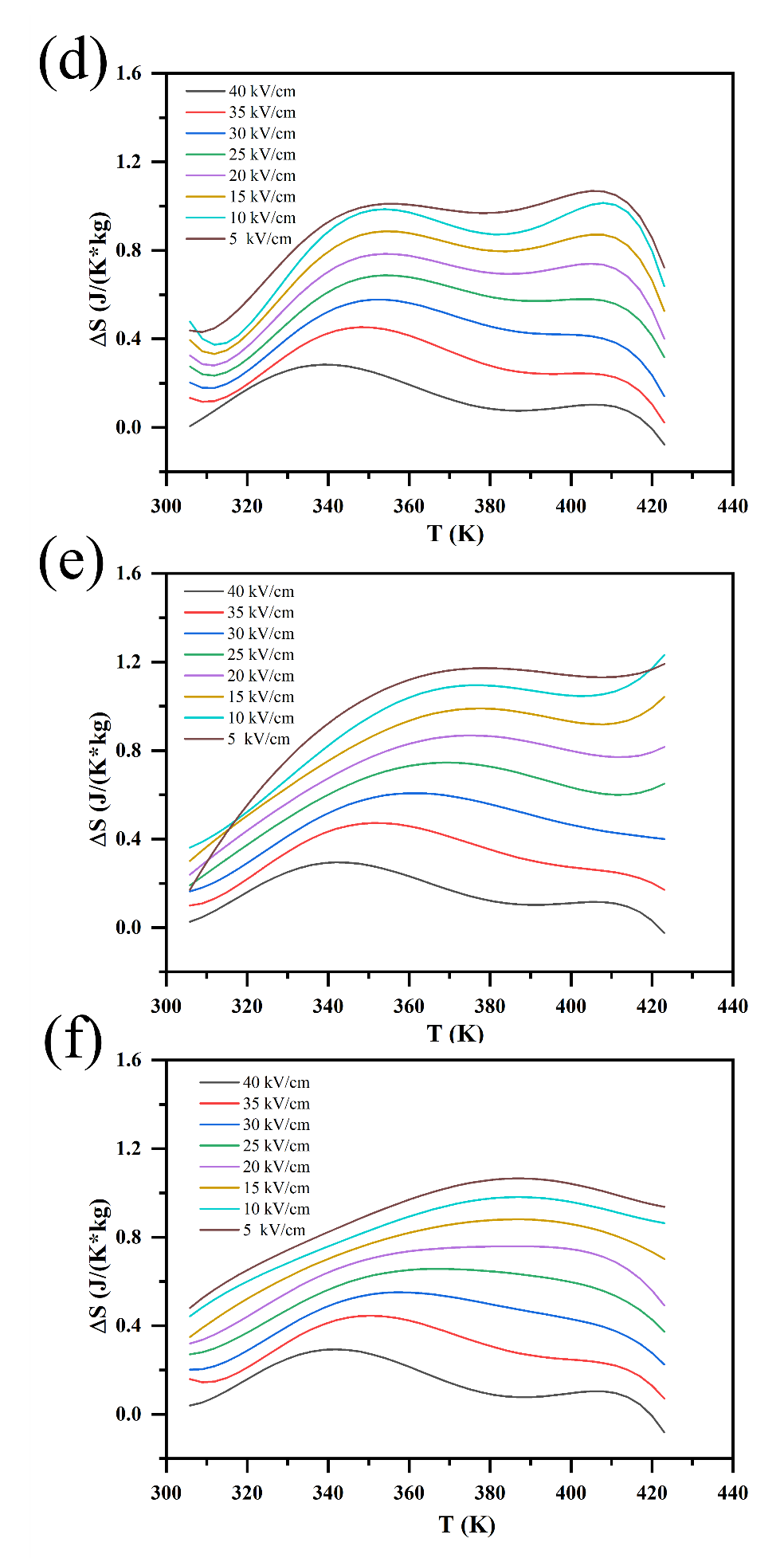


Fig. S9. The relationship between the entropy variation ΔS and the temperature (T) for different quenching times (a) x = 0 h, (b) x = 10 h, (c) x = 20 h, (d) x = 30 h, (e) x = 40 h, and (f) x = 50 h.


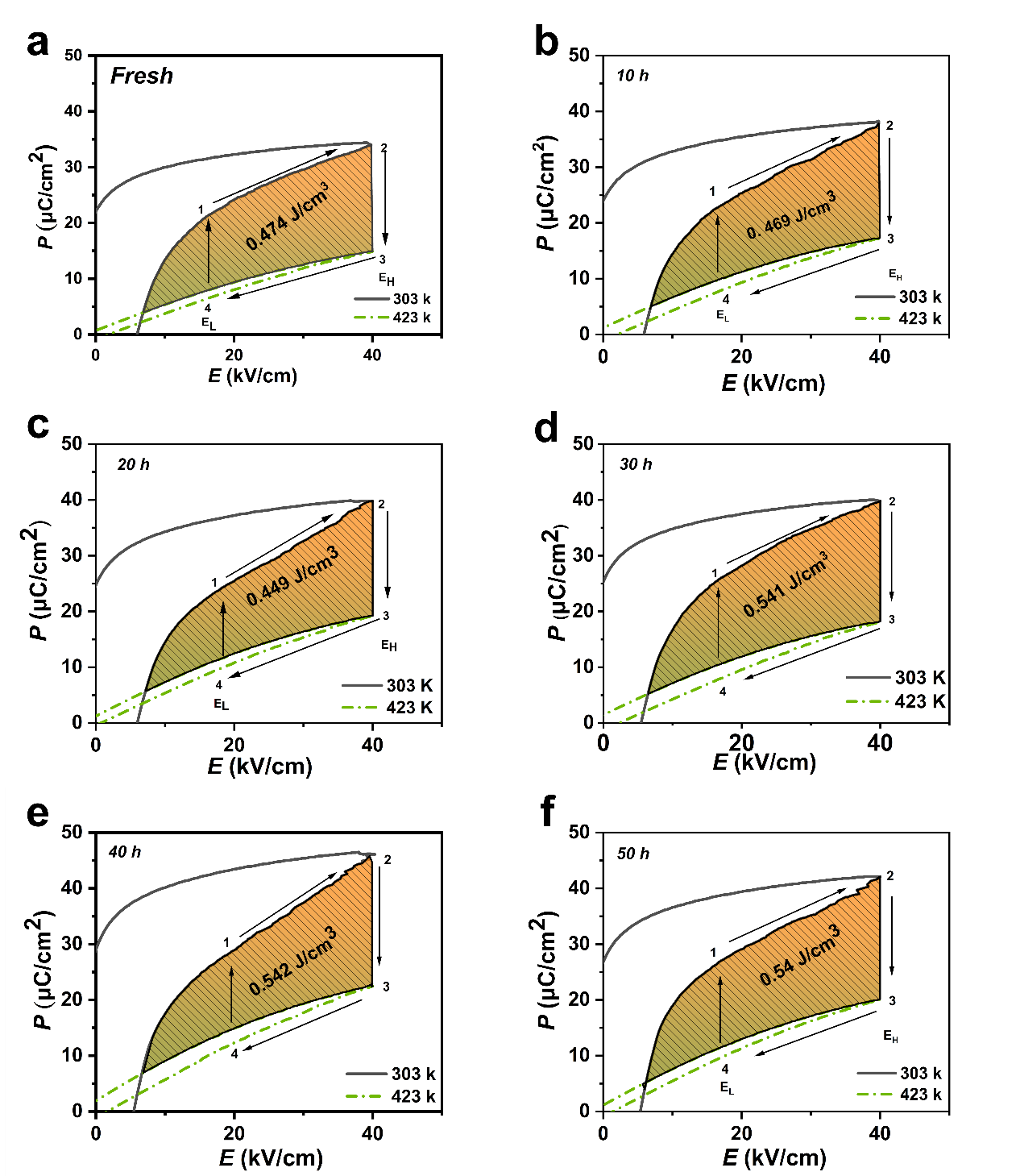


Fig. S10. Olsen cycle diagram of pyroelectric energy harvesting of the PSTT ceramics for different quenching times (a) x = 10 h, (b) x = 20 h, (c) x = 30 h, (d) x = 40 h, (e) x = 50 h, and (f) x = 60 h.


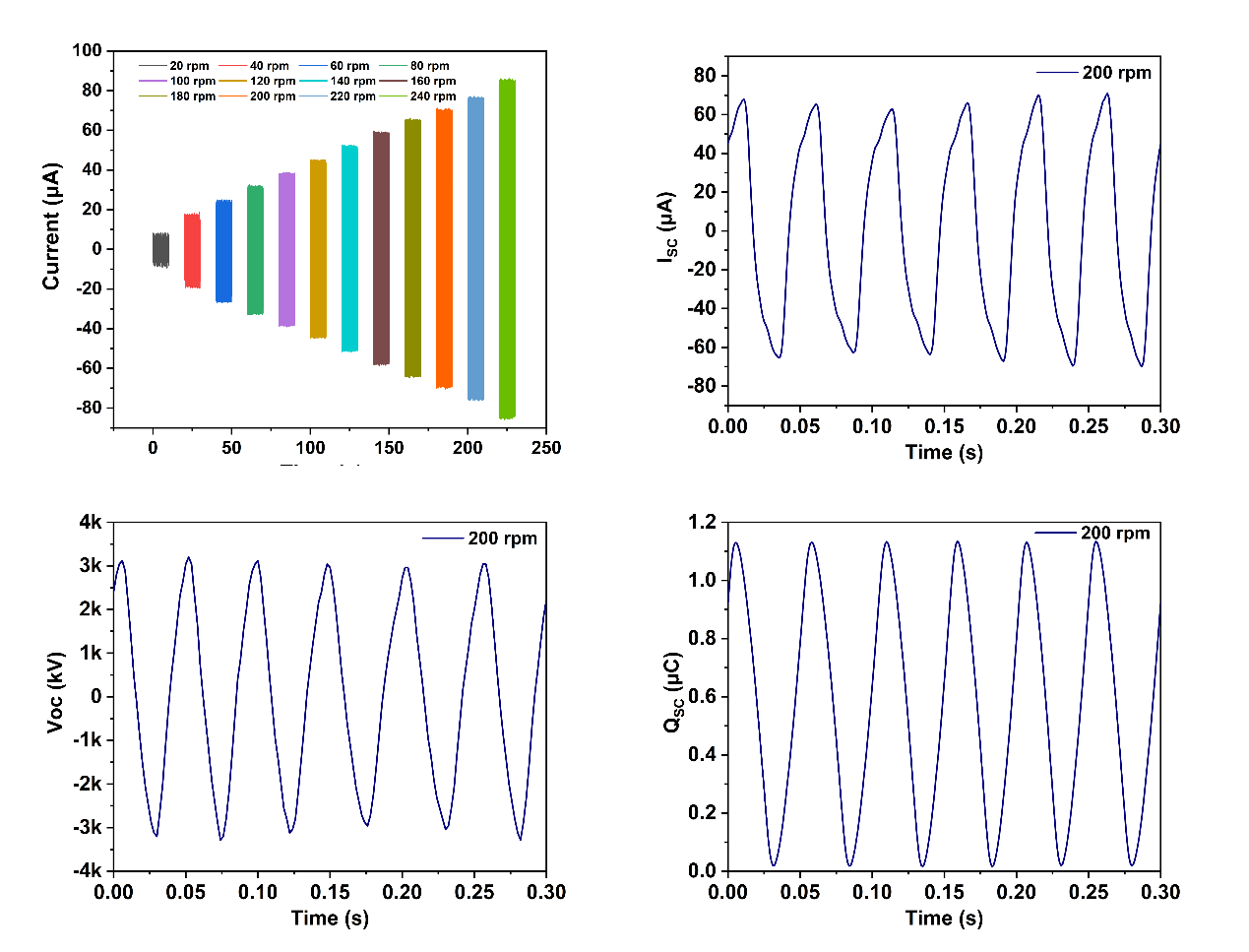


Fig. S11. (a) Short circuit current of TENG at different rotational speeds; (b) short-circuit current, (c) open-circuit voltage, and (d) enlarged view of transferred charge of the TENG at 200rpm.


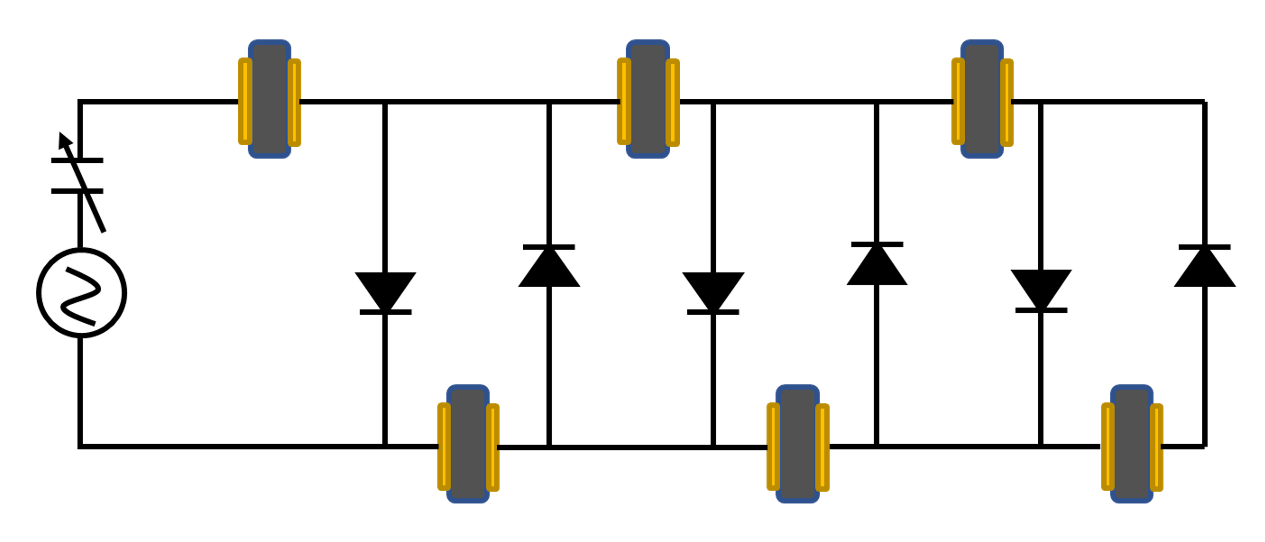


Fig. S12. Circuit simulation of thermostat


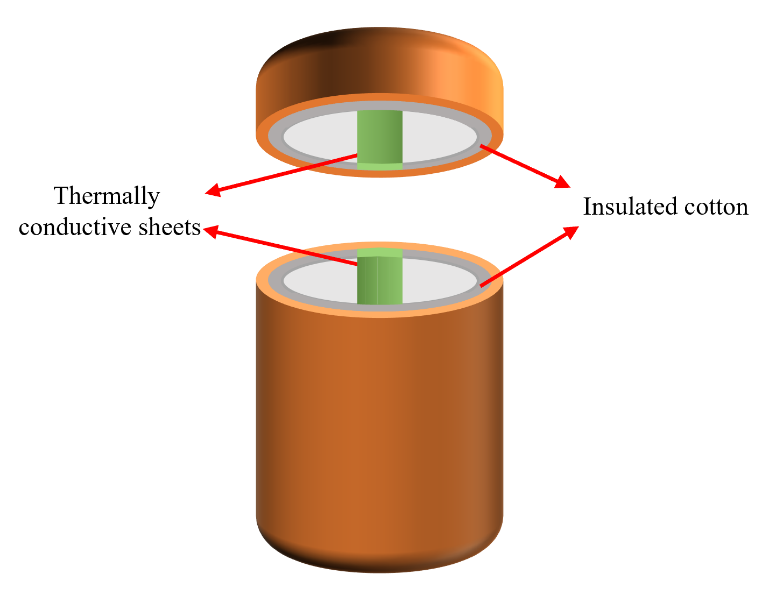


Fig. S13. The internal structure of the SPT 2.0 insulated cup.


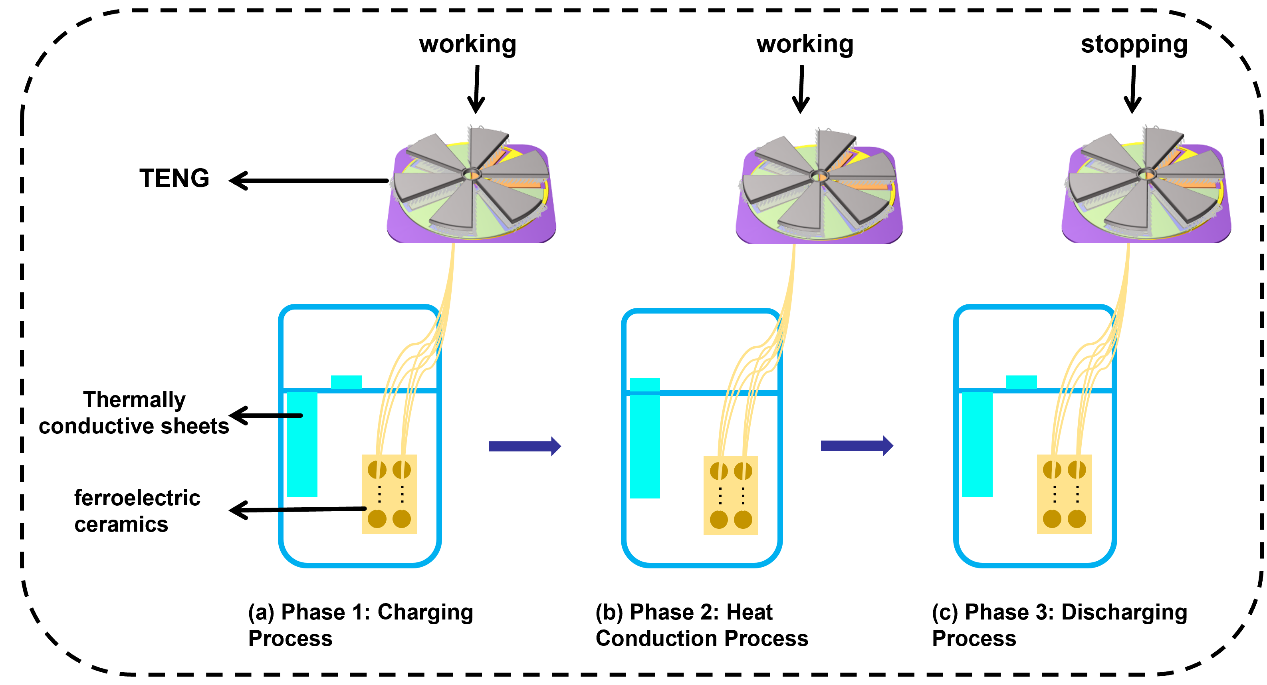


Fig. S14. The SPT 2.0 heating and cooling process is executed via a three-phase mechanism. Initially, in Phase I, the heat conduction plates are detached, preserving the insulating environment of the thermos. The Triboelectric Nanogenerator (TENG) continuously applies an electric field to the ceramic plate inside the cup, reducing its entropy and causing the temperature to rise steadily. After a period, the temperature inside the cup stabilizes. Upon reaching a steady state, the cup lid is rotated to connect the heat conduction plates on the cup body and the lid, initiating Phase II. This action gradually lowers the temperature inside the cup to near ambient levels. Once the temperature stabilizes, the TENG is deactivated and the heat conduction plates are separated, advancing to Phase III. The ceramic plate then begins to discharge its electric field, with the electric field at its ends diminishing progressively. The system's entropy increases, leading to a further decrease in temperature around ambient levels until the electric field at the ceramic plate's ends reaches zero. At this point, the system's temperature is lower than room temperature and becomes stable.


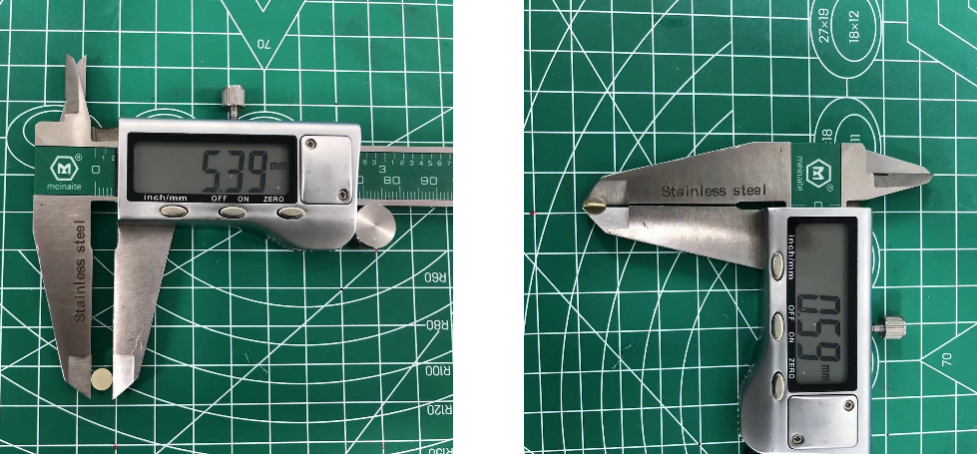


Fig. S15. Diameter (left) and thickness (right) of 0.15PT-0.85PST ceramic sheet.
